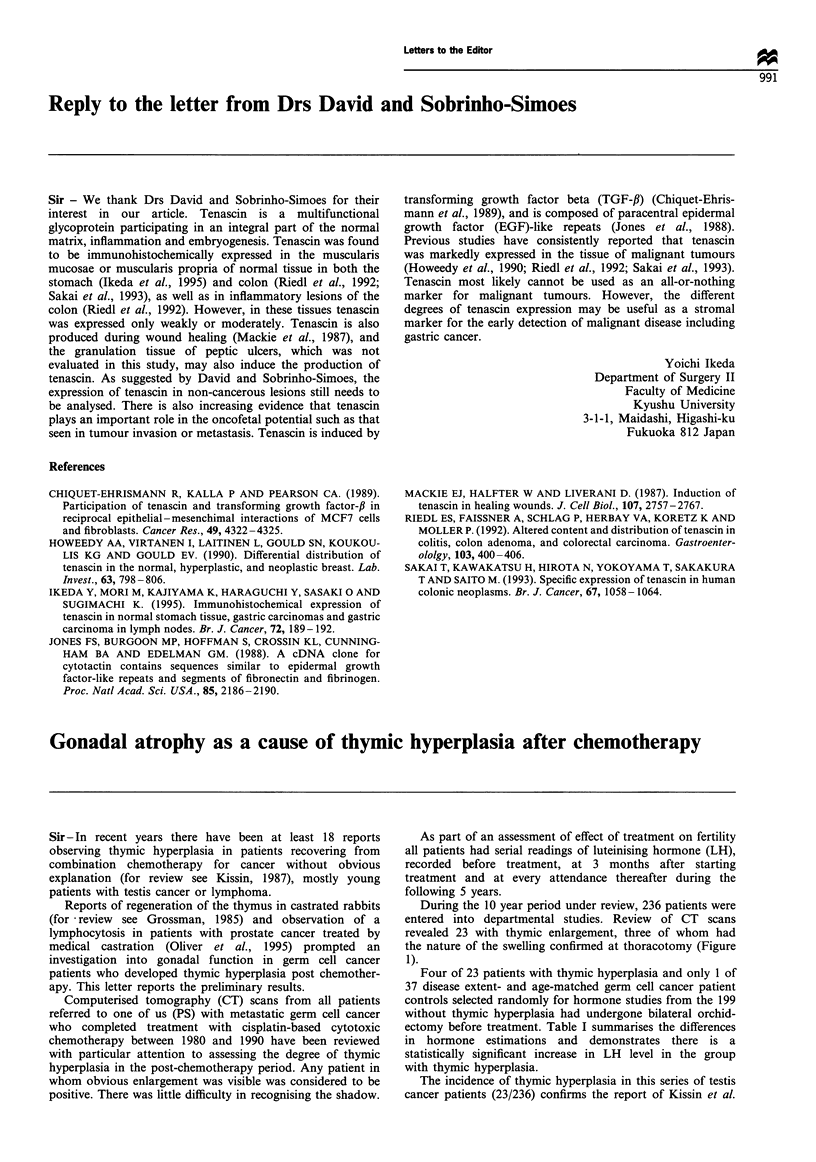# Reply to the letter from Drs David and Sobrinho-Simões

**Published:** 1996-09

**Authors:** Yoichi Ikeda


### Reply to the letter from Drs David and Sobrinho-Simoes

Sir – We thank Drs David and Sobrinho-Simoes for their interest in our article. Tenascin is a multifunctional glycoprotein participating in an integral part of the normal matrix, inflammation and embryogenesis. Tenascin was found to be immunohistochemically expressed in the muscularis mucosae or muscularis propria of normal tissue in both the stomach (Ikeda *et al.*, 1995) and colon (Riedl *et al.*, 1992; Sakai *et al.*, 1993), as well as in inflammatory lesions of the colon (Riedl *et al.*, 1992). However, in these tissues tenascin was expressed only weakly or moderately. Tenascin is also produced during wound healing (Mackie *et al.*, 1987), and the granulation tissue of peptic ulcers, which was not evaluated in this study, may also induce the production of tenascin. As suggested by David and Sobrinho-Simoes, the expression of tenascin in non-cancerous lesions still needs to be analysed. There is also increasing evidence that tenascin plays an important role in the oncofetal potential such as that seen in tumour invasion or metastasis. Tenascin is induced by transforming growth factor beta (TGF-$\beta$) (Chiquet-Ehrismann *et al.*, 1989), and is composed of paracentral epidermal growth factor (EGF)-like repeats (Jones *et al.*, 1988). Previous studies have consistently reported that tenascin was markedly expressed in the tissue of malignant tumours (Howeedy *et al.*, 1990; Riedl *et al.*, 1992; Sakai *et al.*, 1993). Tenascin most likely cannot be used as an all-or-nothing marker for malignant tumours. However, the different degrees of tenascin expression may be useful as a stromal marker for the early detection of malignant disease including gastric cancer.

Yoichi Ikeda
Department of Surgery II
Faculty of Medicine
Kyushu University
3-1-1, Maidashi, Higashi-ku
Fukuoka 812 Japan

**References**

CHIQUET-EHRISMANN R, KALLA P AND PEARSON CA. (1989). Participation of tenascin and transforming growth factor-$\beta$ in reciprocal epithelial-mesenchimal interactions of MCF7 cells and fibroblasts. *Cancer Res.*, **49**, 4322-4325.

HOWEEDY AA, VIRTANEN I, LAITINEN L, GOULD SN, KOUKOULIS KG AND GOULD EV. (1990). Differential distribution of tenascin in the normal, hyperplastic, and neoplastic breast. *Lab. Invest.*, **63**, 798-806.

IKEDA Y, MORI M, KAJIYAMA K, HARAGUCHI Y, SASAKI O AND SUGIMACHI K. (1995). Immunohistochemical expression of tenascin in normal stomach tissue, gastric carcinomas and gastric carcinoma in lymph nodes. *Br. J. Cancer*, **72**, 189-192.

JONES FS, BURGOON MP, HOFFMAN S, CROSSIN KL, CUNNINGHAM BA AND EDELMAN GM. (1988). A cDNA clone for cytotactin contains sequences similar to epidermal growth factor-like repeats and segments of fibronectin and fibrinogen. *Proc. Natl Acad. Sci. USA.*, **85**, 2186-2190.

MACKIE EJ, HALFTER W AND LIVERANI D. (1987). Induction of tenascin in healing wounds. *J. Cell Biol.*, **107**, 2757-2767.

RIEDL ES, FAISSNER A, SCHLAG P, HERBAY VA, KORETZ K AND MOLLER P. (1992). Altered content and distribution of tenascin in colitis, colon adenoma, and colorectal carcinoma. *Gastroenterololgy*, **103**, 400-406.

SAKAI T, KAWAKATSU H, HIROTA N, YOKOYAMA T, SAKAKURA T AND SAITO M. (1993). Specific expression of tenascin in human colonic neoplasms. *Br. J. Cancer*, **67**, 1058-1064.